# Supplementary material for: Prevalence of antibodies against dengue virus in the city of Buenos Aires: Results of a probabilistic population survey
Source: PLoS Negl Trop Dis. 2025 Feb 18;19(2):e0012886. doi: 10.1371/journal.pntd.0012886 (PMC11856294; doi:10.1371/journal.pntd.0012886)
Supplement: Attachment — Submitted filename: Response to reviewers.docx [file pntd.0012886.s002.docx]

**Response to reviewers**

**Reviewer # 1**

**Comment #1**: The authors describe their study to determine the prevalence of dengue virus (DENV) antibody in adult residents after the 2023 season and before the beginning of a dengue outbreak in 2024 in Buenos Aires Argentina. A survey was conducted among adults 18 years and older who resided in the city of Buenos Aires with an adult population of 2.38 million people. Blood samples were tested for DENV IgG and IgM antibodies using a rapid chromatographic immunoassay. If positive for antibody, the results were confirmed using an enzyme-linked fluorescence assay (ELFA). Out of a sampling frame of 2,998 selected households, the interviewers visited 100% of the residences. The overall response rate was 26.8%. Weighted estimates indicated that a total of 193,707 participants had DENV antibodies. implying a weighted antibody prevalence of 8.12%. The prevalence rate varied with participants in the slums having a rate twice as high (14.7%) as those in the rest of the city (7.67%). The findings indicated that adults in Buenos Aires had experienced a moderate amount of DENV infections, but according to the authors the amount was under the thresholds usually considered for implementing a mass vaccination campaign and the results underscored the need for frequent surveys.

The rationale for this study was justified by addressing DENV as a global public health concern that is the most frequent cause of human disease of all the mosquito-borne viruses and globally causes an estimated 100–400 million infections, including severe and fatal cases each year. Therefore, the contents of this manuscript are highly appropriate for publication consideration in this journal. However, the suggested comments should be considered for improving the contents of the manuscript

**Response # 1**: Thank you for your interest and for the overall evaluation of our work. We will now proceed to address each of your comments

**Comment #2**: The objective in the abstract should include a brief description of the burden of dengue disease in the population for justifying the decision to perform the survey. The methods in the abstract state that a stratified, multi-stage probabilistic population survey was conducted that should be described for the readers who do not understand and the reasons for selecting this survey method, may be best to describe in the method section.

**Response #2**: We have added the following sentence to the abstract (lines 24 to 26): Dengue is a rapidly growing global health issue. In Argentina, the disease burden is primarily based on case reports or retrospective series; however, there are no prospective probabilistic studies available.

We agree that additional details on the stratified, multi-stage probabilistic survey methodology and the rationale for its selection would enhance the clarity of our manuscript. In the Abstract, we aim to maintain brevity, but we clarified the methodology by rephrasing it as follows: "A stratified, multi-stage probabilistic population survey was conducted to ensure representativeness of the adult population of Buenos Aires, considering geographical and socio-economic diversity." (lines 32 to 34).

The details of the stratified, multi-stage probabilistic survey are already described comprehensively in the Methods section. Specifically, we provide information on the sampling frames, stratification criteria, and the multi-stage sampling process, including the selection of primary units, dwellings, and participants within households. Additionally, we explain the rationale for using this method, which ensures representativeness of the city’s diverse population and reduces sampling bias. However, we acknowledge that readers less familiar with these methodological concepts might benefit from further clarification. To address this, we have added the following explanation to the Methods section: “A stratified, multi-stage probabilistic survey was chosen as it is widely used in epidemiological studies to ensure representativeness of a heterogeneous population. This approach allows for the inclusion of diverse subpopulations by dividing the sampling frame into strata based on relevant criteria, such as income levels and geographic areas. By employing multi-stage sampling, we optimize resource allocation while maintaining statistical reliability, as each stage progressively narrows the selection from broader units (e.g., census tracts) to individual households and participants. This methodology reduces sampling bias and enhances the validity of results for an urban area as complex and diverse as Buenos Aires.” (lines 139 to 145).

**Comment #3**: Why were only adults selected for inclusion in this survey, again can be done in the method section?

**Response#3**: Thank you for your comment. The decision to include only adults in this survey was based on logistical considerations. To address your concern, we have added the following explanation to the strengths and limitation section: This survey focused on adults (aged 18 years and older) due to logistical and regulatory constraints. In Argentina, including children in research studies presents significant challenges, as it requires obtaining parental consent and navigating complex ethical and regulatory processes. Additionally, practical considerations, such as coordinating with schools or other institutions, would have made the study substantially more difficult to implement within the available timeframe and resources. For these reasons, the study was limited to the adult population, while acknowledging that the findings may have limited generalizability to younger age groups (lines 443 to 450).

**Comment #4**: Interviews are mentioned in the results section of the abstract but are not mentioned in the methods, again can be done in the method section as well as the sampling frame should be defined as well as households, did this include all adults over 18 years of age (2.38 million) except those excluded by the eligibility criteria.

**Response #4**: Thank you for pointing out the need to clarify the description of interviews and the sampling frame. To address this, we have included the following additions to the Methods section (lines 230-232): "The interviews were conducted using a structured questionnaire designed to collect demographic information, medical history, and knowledge of dengue. The questionnaire was pre-tested in a pilot study to ensure clarity and reliability. Regarding the sampling frame, we have revised the text (lines 151-178).

**Comment #5**: The methods in the abstract, or in the method section do not state how many participants were actually enrolled in the survey other than stating that the survey was conducted among adults (selected areas and households) residing in the city of Buenos Aires, representing the city’s 2.38 million adult population. An actual number of the participants would be more informative.

**Response #5**: The total number of participants enrolled in the survey (804) is already stated in the Results section. However, we understand the importance of making this information more immediately visible. To address this, we propose including the number of participants in both the Abstract and the Methods section for improved clarity and accessibility.

Particularly, in the abstract section we modified the phrasing to: A total of 804 participants were enrolled, representing the city’s 2.38 million adult population (lines 46 to 47).

On the other hand, in the results section, we added: Out of the 2,998 selected households, 804 individuals participated in the study, resulting in a response rate of 26.8%. Weighted estimates were used to adjust for non-response and to provide prevalence rates representative of the total adult population of Buenos Aires (2.38 million). (lines 269 to 272).

Please also note that Table 1 presents the unweighted absolute number for each characteristic and group (line 483).

**Comment #6**: While the antibody assay techniques are stated, nothing is mentioned as to what samples were tested, assume paper adsorbed blood samples for antibody or how the techniques were performed, assume in accordance to the manufacturer’s instructions and what dilution(s) of the sample were tested, again may be done in the method section.

**Response #6**: We have added the following clarifications to the Methods section: The samples tested consisted of capillary blood obtained through a finger-prick. Blood was directly applied to the test strip of a rapid immunochromatographic assay, following the manufacturer’s instructions. The assay does not require additional dilution of the blood sample, as it is designed to analyze whole blood directly (lines 236 to 240).

For participants with positive results, venous blood samples were collected at the Virology Unit of the F.J. Muñiz Hospital and processed for IgG antibody detection using the VIDAS® Anti-Dengue IgG (DENV) (bioMérieux) assay. We have added the following clarification to the Methods section (lines 243-259): For individuals with positive IgG results in the rapid test, venous blood samples were collected and processed at the Virology Unit of the Hospital F.J. Muñiz. Serum was obtained by centrifugation of the blood samples and analyzed using the VIDAS® Anti-Dengue IgG assay (bioMérieux), which employs the ELFA (enzyme-linked fluorescence assay) technique. The assay was performed following the manufacturer’s instructions, which include an automatic dilution step by the VIDAS® system. This involves mixing the serum sample with reagents and applying it to a solid-phase device coated with recombinant dengue antigens. The fluorescence intensity measured at the end of the process is proportional to the concentration of IgG antibodies present in the sample. No manual dilution of the serum was performed. Controls for the rapid test included manufacturer-provided positive and negative samples, which were run during each testing session. The VIDAS® Anti-Dengue IgG assay was selected for confirmation due to its higher specificity (99.1%) and semi-quantitative design, which complements the qualitative nature of the rapid test

**Comment #7**: The content of the introduction section could be improved by a brief description of the global dengue disease burden as well as the burden of the disease that outbreaks have caused in Buenos Aires. Also, the contents of the introduction section is focused on dengue vaccines with nothing much included on history of dengue in Buenos Aires, including the ecology and epidemiology of dengue in Buenos Aires and other communities in Argentina.

**Response #7**: We do now include a brief overview of the global dengue burden and additional details on the history, ecology, and epidemiology of dengue in Buenos Aires. To address this, we propose the following modifications: In Buenos Aires, dengue re-emerged at the end of the 1990. Since then, outbreaks have occurred intermittently, with increasing intensity and frequency. The most significant outbreak occurred in 2023. This outbreak was characterized by the co-circulation of DENV-1 and DENV-2, the latter being predominantly of the Cosmopolitan genotype. (Lines 91 to 94). Also we added: The ecology of dengue in Buenos Aires is influenced by urbanization, climate variability, and the widespread presence of Aedes aegypti, the primary mosquito vector. High population density and unplanned urban growth, particularly in informal neighborhoods, create ideal conditions for vector proliferation and dengue transmission (lines 95 to 99)

**Comment #8**: Also, if surveillance for dengue cases is being done, this should be described and what is being done to prevent dengue cases, such as vector control. As the author’s knows, the majority of DENV infections are asymptomatic and therefore, is a weakness of serosurveys for public health purposes as antibody prevalence rates only indicate prior infections and nothing about disease, and also is limited for assessing current infection rate because antibody can represent infections that occurred many years ago. However, the information is of public health value for monitoring the status of the potential burden of disease and for estimating the efficacy of vector and/or vaccine control measures.

**Response # 8**: We acknowledge the importance of describing the ongoing surveillance efforts and preventive measures for dengue in Buenos Aires. To address this, we have added the following information:

Dengue surveillance in Buenos Aires relies on a mandatory notification system integrated into the national epidemiological framework. Cases are identified through a combination of clinical criteria and laboratory confirmation, which include PCR, NS1 antigen detection, and serology. Vector control measures implemented by the city's public health authorities include regular fumigation in high-risk areas, education campaigns targeting mosquito breeding sites, and community participation to eliminate standing water. These measures aim to reduce Aedes aegypti populations, although their effectiveness is influenced by environmental and socio-economic factors (lines 106 to 113)

Regarding the limitations of serosurveys we have added the following clarification to the Limitations section: Seroprevalence surveys provide valuable insights into the cumulative exposure of a population to dengue virus (DENV), but they have inherent limitations. Most DENV infections are asymptomatic, and antibody detection reflects prior exposure rather than current infection rates. This means that seroprevalence data cannot distinguish between recent and past infections, potentially obscuring short-term epidemiological trends. Despite this, serosurveys remain an essential tool for public health planning, as they help monitor the long-term burden of disease, identify high-risk populations, and evaluate the impact of vector control and vaccination programs. In Buenos Aires, these data complement surveillance systems, which are focused on detecting symptomatic cases, and vector control measures aimed at reducing Aedes aegypti populations (lines 451 to 460).

**Comment #10**: Additional comments to improve the manuscript pertain to the study area that needs to be describe especially the slums versus the non-slum areas of the city to include any relevant information on Aedes aegypti and/or Aedes albopictus.

**Response #10**: Thank you for your comment highlighting the need to better describe the study area and include information on the Aedes vectors. While we have already addressed these aspects in the Methods and Introduction sections, we recognize the importance of emphasizing the unique characteristics of slum and non-slum areas and their relevance to dengue transmission. To provide further clarity, we have included the following additions:"

In the introduction section we now added: Slums, representing approximately 7% of the city's dwellings, are characterized by poor infrastructure, inadequate water and sanitation services, and higher vulnerability to Aedes aegypti proliferation. In contrast, non-slum areas generally benefit from better urban infrastructure. Although Aedes aegypti is the predominant vector in Buenos Aires, Aedes albopictus has not been documented in the city, likely due to climatic and ecological factors (lines 99 to 105).

In the methods section: These areas are distinct in their socio-economic conditions and environmental risks, which influence mosquito breeding and dengue transmission dynamics. (lines 172 to 174).

In the discussion section: The higher seroprevalence observed in slums compared to non-slum areas is consistent with the ecological and socio-economic disparities described in the literature. While Aedes albopictus is not currently present in Buenos Aires, continuous monitoring is essential given the potential for environmental changes to alter vector distribution (lines 366 to 370)

**Comment #11**: It is stated that the selected dwellings were visited by interviewers and nurses, who randomly selected one person to complete the survey. What was the instrument used to perform the interviews and who was the one person selected to participate in the interviews? While the sampling procedure is described, it is not clear as to how reliable the estimated prevalence rate was for the entire city. If the assumption was made that the rate based on a sample of the population would be applicable to the total population, this would need to account for the well established understanding that DENV infection is not randomly distributed but occurs in clusters based on the risk associated with the present and movement of people and places where contact with infected mosquitoes are likely that causes marked heterogeneity in transmission rates in a community.

**Response #11**: As described in the Methods section, one adult (aged 18 or older) was randomly selected from each household using the Kish method, which ensures unbiased selection among eligible household members. The interviews were conducted using a structured questionnaire designed to collect demographic information, medical history, and knowledge of dengue. The questionnaire was pre-tested in a pilot study to ensure clarity and reliability. This information has been further detailed in the revised Methods section (lines 230-232).

The prevalence estimates in this study were calculated using a stratified, multi-stage probabilistic sampling design, which is standard for population-based surveys. This method accounts for the heterogeneity in dengue transmission across different strata, such as slums versus non-slum areas. Weighted estimates were used to adjust for non-response and ensure representativeness of the city’s 2.38 million adult population. However, as the reviewer correctly notes, dengue transmission is highly heterogeneous and influenced by factors such as mosquito density, human movement, and socio-economic conditions. This clustering effect introduces inherent variability in prevalence estimates. To address this, we have included a note in the Limitations section acknowledging the potential impact of spatial heterogeneity on our results and the importance of complementing serosurveys with spatial analysis to better capture these dynamics: Dengue transmission is known to occur in spatial clusters, influenced by local vector density, environmental factors, and human movement patterns. While our sampling design aimed to capture this variability by stratifying the population and using weighted estimates, the clustering effect may lead to underestimation or overestimation of prevalence in certain areas. Future studies should incorporate geospatial modeling to better understand and address this heterogeneity (lines 461 to 466)

**Comment #12**: The methods used to perform the assay to test samples for antibody needs to be described in more detail, for examples, in addition to the comments mentioned in the abstract, were controls run in the tests, including antibody positive and negative samples to validate the performance of the test. Although the test according to the manufacturer has a very high sensitivity and specificity, confirmation of these test parameters should have been done prior to use in this study. A second test was mentioned for use to confirm the results of the first test, why did the results of the first test need to be confirmed and what was different about the second test that supported it use as a confirmation assay. Also, the very low samples available from only 27 participants for confirmation by a second test present some concerns regarding the validity of the results of this study. Overall, the results are adequately described, but could be improved by more detailed description to clarify some of the observations.

**Response #12**: As part of the laboratory procedures, positive and negative control samples provided by the manufacturer were run during each testing session to validate the performance of the rapid immunochromatographic assay (Laboratorios JAYOR SRL). Additionally, internal quality control measures, including technician training and supervision, were implemented to ensure consistent and reliable results. These details have been added to the Methods section: Controls for the rapid test included manufacturer-provided positive and negative samples, which were run during each testing session. The VIDAS® Anti-Dengue IgG assay was selected for confirmation due to its higher specificity and semi-quantitative design, which complements the qualitative nature of the rapid test (lines 252-256)."

The performance characteristics of the rapid test, as reported by the manufacturer (sensitivity: 94.3%, specificity: 99.1% for IgG), were cross-referenced with published evaluations of the test in similar settings. While no formal validation was conducted prior to this study, the test’s parameters were deemed suitable for use based on its reported concordance with ELISA in peer-reviewed studies.

The second test, VIDAS® Anti-Dengue IgG (bioMérieux), was used to confirm positive IgG results due to its higher specificity and semi-quantitative capabilities, which allow for better differentiation of antibody levels. This approach was implemented to address the potential for false positives inherent to rapid tests and to strengthen the validity of the findings. These clarifications have been added to the Methods section: (lines 243-256)."

We acknowledge the concern regarding the low number of samples confirmed by the second test. This limitation was primarily due to logistical and resource constraints, as well as participant non-compliance with follow-up appointments for venous blood draws. Despite this, the high concordance (100%) between the rapid test and the VIDAS® assay for the confirmed samples supports the reliability of the rapid test in this population. We have expanded the Limitations section to explicitly address this issue and its potential impact on the study’s conclusions: The proportion of participants who underwent confirmatory testing was limited (27/71), which may affect the generalizability of the confirmatory results. However, the 100% concordance observed supports the reliability of the rapid test in this context (lines 435 to 438)

**Comment #13:** Only 26.8% of 2,998 selected households were interviewed, did this low rate provide data representative of the total study population and was the 26.8% included as participants of this study. The number of participant tested is stated as a total 193,707 people aged 18 and over tested positive for DENV antibodies. To clarify the reviewer’s previous comment on participants in the study, how many participants were enrolled, how many were tested and how many were positive of the total participants tested. The results presented in Table 1 need to be described more-so in the results and discussion sections. For example, the age distribution of antibody positive participants decreased from 51.6% for the 18-34 ages to 25.5% for the 65 and over ages. In a dengue endemic community, like Buenos Aires, especially being a hyperendemic community, the antibody prevalent rate would be expected to be the reverse indicating that the individuals who lived the longest in the community would have the highest antibody prevalent rate. Also, the overall detection of DENV antibody in about 8% of the population is not considered to be a moderate prevalent rate, but a rate that indicate a low risk for DENV infection

**Response #13**: As stated in the Results section, 26.8% of the 2,998 selected households completed the survey, corresponding to 804 individual participants. These participants were enrolled and tested as part of the study. Weighted estimates were applied to ensure that the results were representative of the total adult population of Buenos Aires, accounting for non-response and stratification by socio-economic and geographic factors. We have clarified this in the Methods section: Out of the 2,998 selected households, 804 individuals participated in the study, resulting in a response rate of 26.8%. Weighted estimates were used to adjust for non-response and to provide prevalence rates representative of the total adult population of Buenos Aires (2.38 million) (lines 269 to 272)

A total of 804 participants were tested for DENV antibodies, of which 71 tested positive (8.1%) based on the weighted prevalence estimate of 193,707 adults in the population. This clarification has been added to the Results section: A total of 804 participants were tested for DENV antibodies. Based on these results, 71 individuals tested positive, corresponding to a weighted prevalence of 8.12% [95% CI: 8.08%-8.15%], equivalent to approximately 193,707 adults in the city’s population (lines 282 to 284)

We acknowledge the reviewer’s comment regarding the age distribution of antibody-positive participants. The observed trend of decreasing seroprevalence with increasing age, from 51.6% in the 18–34 age group to 25.5% in those aged 65 and older, contrasts with patterns seen in hyperendemic settings. In Buenos Aires, this likely reflects the introduction of dengue in the late 1990s, with younger age groups experiencing higher exposure during recent outbreaks. We have expanded the Discussion section to reflect this observation and its implications: The age distribution of DENV antibody-positive participants showed a higher prevalence in younger adults compared to older age groups . This pattern contrasts with hyperendemic areas, where cumulative lifetime exposure leads to higher seroprevalence in older individuals. In Buenos Aires, the lower prevalence in older adults likely reflects the reintroduction of dengue in the late 1990s and the concentration of more recent outbreaks among younger, more mobile populations. This trend underscores the need for targeted prevention strategies and ongoing surveillance to monitor shifting transmission dynamics (lines 359 to 366)

We appreciate the reviewer’s perspective on the interpretation of the overall seroprevalence rate (8.1%). While this rate is indeed low compared to hyperendemic areas, we chose to describe it as moderate given the relatively recent reintroduction of dengue in Buenos Aires and the city’s susceptibility to periodic outbreaks. To address this, we have rephrased the Discussion to acknowledge the low endemicity while highlighting its significance for public health planning. We added in the discussion the following statement: The overall seroprevalence indicates a low level of endemicity for dengue in Buenos Aires, consistent with its history of recent reintroduction and episodic outbreaks. While this rate suggests a lower force of infection compared to hyperendemic settings, it remains significant for public health planning given the city’s vulnerability to future outbreaks and the potential for increasing transmission intensity (lines 321 to 326).

**Comment #14**: The discussion section tends to focus on dengue vaccines and should address more-so the observations of this study, for example, it is stated that “Although dengue is a notifiable disease, it is likely that the number of reported cases underestimates the disease burden due to the presence of asymptomatic or mildly symptomatic cases, as well as a tendency for underreporting communicable diseases” Not clear as to the meaning of the latter sentence, as asymptomatic cases would not contribute to the burden of disease, under-reporting would lessen the estimated burden of disease, but is there evidence for under-reporting of cases. In regard to the burden of disease associated with dengue, estimates discussed are based on ≥18 years for 2007, why do estimated not include all ages especially with dengue being more of a pediatric disease. In contrast to estimates in Buenos Aires at 682/100,000, the results of this study suggested a disease burden of 9,050 cases per 100,000 inhabitants, how reliable is the latter estimates and what is the explanation for the difference. According to the authors, the disparity between reported cases and disease burden as measured by seroprevalence indicates that many dengue infections are asymptomatic, and the cases reported through surveillance systems represent only a small fraction of the actual cases. The reviewer agrees that the majority of DENV infections is asymptomatic, then using seroprevalence data is not a reliable estimate of the burden of disease, whereas cases reported by passive surveillance will detect only the more severe cases, while an active surveillance will provide the best estimate of the number of cases, thus the best estimate of the burden of disease. Therefore, since the outcome of DENV infection cannot be determined based seroprevalence estimates, the reviewer suggest that an explanation be provided regarding their statement that serial population based seroprevalence studies can serve as a robust system for assessing disease burden.

**Response #14**: We appreciate the reviewer’s comments and the opportunity to clarify our interpretation of disease burden. While we agree that seroprevalence estimates alone cannot directly measure the clinical burden of disease, we argue that they provide critical insights into the epidemiological burden. In vector-borne diseases like dengue, seropositive individuals, even if asymptomatic, contribute to the overall risk in the population by serving as potential reservoirs for the virus. This role underscores the importance of seroprevalence studies not only in assessing past exposure but also in evaluating population-level susceptibility and the potential for future outbreaks. To reflect this perspective, we have revised the Discussion section as previously commented.

We acknowledge the reviewer’s point regarding the exclusion of pediatric populations in this study. Our estimates were limited to individuals aged 18 and older due to logistical and regulatory constraints, as detailed in the Methods section. While dengue disproportionately affects pediatric populations in endemic settings, this study focused on the adult population of Buenos Aires, where the disease has a distinct epidemiological profile (please see response #3).

The disparity between reported cases (682/100,000) and seroprevalence-based estimates (9,050/100,000) underscores the underreporting inherent in passive surveillance systems, which primarily detect moderate to severe cases. Yet seroprevalence does provides valuable insights into historical transmission dynamics, population immunity, and susceptibility. Serial population-based seroprevalence studies, when combined with active surveillance, can strengthen disease monitoring by capturing changes in transmission intensity and identifying high-risk populations. We added the following sentence in the discussion: However, it should also be noted that while seroprevalence cannot directly measure the clinical burden of dengue, it serves as a complementary tool to active and passive surveillance. By identifying immunity gaps and historical exposure, serial seroprevalence studies provide baseline data critical for modeling future outbreaks, guiding vaccination strategies, and evaluating vector control measures. However, their use should always be contextualized within a broader surveillance framework (lines 349 to 354).

**Comment #15**. Specific comments:

The grammar need to be corrected mainly to for the use of past tense, the following are examples:

Lines 36 -37. “The overall response rate was 26.8%. Weighted estimates show a total of 193,707 people aged 18 and over have antibodies to DENV” (the “word” show should be “showed”). The sentence was modified according to the reviewer’s suggestion (line 48)

Lines 149-151. The 149 performance parameters of this test, as reported by the manufacturer, indicate (the word” indicate” should be “indicated”) a sensitivity of 95.8%, 95% CI: 91.1-98.4%, and a specificity of 96.1%, 95% CI: 92.6- 151 98.4%, compared to ELISA assays. The sentence was modified according to the reviewer’s suggestion (line 241).

Lines 210-211. In contrast, our study suggests (should be suggested) a disease burden of 9,050 cases 211 per 100,000 inhabitants. The sentence was modified according to the reviewer’s suggestion (line 337).

Lines 211-214. The disparity between reported cases and disease burden as measured by seroprevalence indicates (should be indicated) that many dengue infections are asymptomatic, and the cases reported through surveillance systems represent (should be represented ) only a small fraction of the actual cases. The sentence was modified according to the reviewer’s suggestion (line 340).

Lines 226-227. These findings are (replace “are” with “were”) consistent with those reported in the literature. The sentence was modified according to the reviewer’s suggestion (lines 370).

Lines 234-236 According to data from the latest epidemiological bulletin, 235 68% of cases are (replace “are” with “were”) from DEN-2, while 32% are from DEN-1, suggesting that among 236 seropositive citizens there is homotypic immunity to both viruses, especially for DEN-2. The sentence was modified according to the reviewer’s suggestion (line 384).

Lines 24 – 26. To estimate the seroprevalence of antibodies against the dengue virus (DENV) in adult residents of Buenos Aires, at the end of the 2023 season and immediately before the outbreak at the beginning of 2024. What caused the outbreaks and a brief statement of the health impact?.

We added the following sentence: "This outbreak was characterized by the co-circulation of DENV-1 and DENV-2, the latter being predominantly of the Cosmopolitan genotype. The ecology of dengue in Buenos Aires is influenced by urbanization, climate variability, and the widespread presence of Aedes aegypti, the primary mosquito vector. High population density and unplanned urban growth, particularly in informal neighborhoods, create ideal conditions for vector proliferation and dengue transmission (lines 94 to 99).

Lines 30-33 The sentences “The survey assessed the presence of IgG and IgM antibodies using a rapid chromatographic immunoassay. In positive cases, participants were invited for result confirmation through antibody determination using an enzyme-linked fluorescence assay (ELFA) technique” could be improved by considering the following: The survey determined the seroprevalence rate of DENV IgG and IgM antibodies using a rapid chromatographic immunoassay. Antibody positive participants were invited for re-testing to confirm antibody result using an enzyme-linked fluorescence assay (ELFA) technique. The sentence was modified according to the reviewer’s suggestion (lines 36 to 39).

Lines 42-44 states that “Seroprevalence results in adults in Buenos Aires show moderate exposure to the dengue virus (DENV), although still far from the thresholds usually considered for initiating a mass vaccination campaign”, suggest moderate “DENV infection rate” to replace the word, ” exposure” and delete dengue and use only DENV. The sentence was modified according to the reviewer’s suggestion (lines 54 to 56).

Lines 94-95 suggest that this sentence “Buenos Aires is the largest city in Argentina and has a population of 3.1 million of inhabitants, with 2.38 million having 18 or more years” be slightly revised to read with 2.38 million people 18 or more years of age. The sentence was modified according to the reviewer’s suggestion (line 132).

In Table 1, the numerical values should be presented with commons rather than periods, for example 2.192.681 should be 2, 192,681, and for 100,0 should be 100 and for 193.707 should be 193,707/ Characteristics Age groups 18-34 years 595.269 should be 595,269, and for 27,1 should be 27.1 etc. We made all these modifications in table 1 (table 1).

**Reviewer #2.**

**Comment # 1**: Selection of Primary Sampling Units (PSUs): The methodology for selecting PSUs from private households is detailed. However, the manuscript also references the inclusion of the slum population from the 'Informal Popular Neighbourhoods' sampling frame. The method for selecting PSUs from this frame is unclear. The authors should provide a description of how PSUs were selected for this population.

**Response # 1**: Thank you for your observation. We recognize that this aspect requires further clarification.
The 'Informal Popular Neighbourhoods' sampling frame consisted of census tracts from the 2010 National Population, Household, and Housing Census, specifically from slum areas in Communes 1, 4, 7, 8, 9, and 15. Each census tract was considered a PSU, referred to as Primary Sampling Areas (Áreas UP). A three-stage sampling design was implemented:

1. In the first stage, 20 PSUs were selected with probability proportional to size, using the total number of dwellings as the measure of size.
2. In the second stage, 10 dwellings were randomly selected within each PSU.
3. In the third and final stage, one resident from each selected dwelling was chosen using a randomization process to participate in the survey.

This design ensured representativeness of the slum population while maintaining consistency with the probabilistic approach applied to the general private households’ frame. A total of 200 dwellings were included from this sampling frame.

We have included a concise explanation in the Methods section describing the selection process for PSUs in the 'Informal Popular Neighbourhoods: For the 'Informal Popular Neighbourhoods' sampling frame, census tracts from slum areas in Communes 1, 4, 7, 8, 9, and 15 were used as Primary Sampling Units (PSUs). A three-stage sampling design was applied: 20 PSUs were selected with probability proportional to size, 10 dwellings were randomly chosen within each PSU, and one resident was randomly selected per dwelling. This approach ensured representativeness of the slum population within the study (lines 166 to 172).

**Comment #2**: Sample size for the survey: 2. (First paragraph, page 5) Authors selected 3000 dwellings were selected. As per table 1, 2386388 individuals were tested for dengue antibodies. Is this correct? How much is the household/dwelling size in the city. It seems authors applied the seroprevalence estimated from the survey to the entire population of city. I feel Table 1 should only include the number of individuals actually tested and positive and not extrapolation to the entire population

**Response #2**: Thank you for your observation regarding Table 1 and the presentation of data. In population-based probabilistic surveys, it is standard practice to present weighted estimates to ensure the results are representative of the target population. In this study, we calculated weighted seroprevalence estimates to extrapolate our findings to the total adult population of Buenos Aires (2,386,388 individuals). However, we acknowledge the importance of distinguishing raw data from extrapolated estimates for clarity.
Table 1 currently includes weighted estimates to represent the seroprevalence in the total population. This approach is consistent with probabilistic survey methodology and aligns with our study's objective to estimate population-wide seroprevalence. We believe presenting these expanded results provides critical public health insights. To address your concern, we revised the table to include both the raw data (number tested and positive) and the extrapolated estimates, along with a clear explanation in the table footnote (see new table 1 – line 483).

**Comment #3**: Clarification on Calibration by Fixed Margins Methodology: The manuscript refers to using "calibration by fixed margins," following the method developed by Deville and Särndal. As this method might not be familiar to all readers, it would be helpful if the authors could elaborate on how this calibration method was applied. Why did the authors choose this approach over using design weights and sampling methods, as typically recommended in Demographic and Health Surveys (DHS)?

**Response #3**: The calibration by fixed margins method, developed by Deville and Särndal, was used to adjust the initial design weights. This technique minimizes numerical discrepancies between the weighted sample estimates and known auxiliary population totals. In this study, calibration was performed independently for each of the three sampling zones. Auxiliary information used for calibration included population projections from the Buenos Aires Directorate General of Statistics and Censuses (DGESYC) and data from the 2022 Annual Household Survey. The population totals considered were stratified by sex (male, female) and age groups (18–34, 35–49, 50–64, and 65+). This approach was selected because complex sampling designs, such as those used in this study, often involve unequal probabilities of selection, stratification, and clustering, which can lead to under- or over-representation of certain subgroups. Calibration adjusts for these imbalances by incorporating reliable auxiliary information, ensuring that weighted estimates are representative of the target population. This method also respects the original design weights and addresses non-response adjustments effectively.

We added the following sentence in the Methods section: This process used auxiliary data from DGESYC population projections and the 2022 Annual Household Survey, ensuring alignment of the weighted sample estimates with known population totals by sex and age groups. Calibration was performed independently for each of the three sampling zones and included all strata defined in the survey design (lines 203 to 207).

**Comment #4**: Assumptions for Sample Size Estimation: Pl clarify how much was the anticipated prevalence while estimating the sample size

**Response #4**: The sample size estimation assumed an anticipated seroprevalence of 10%, based on historical data and prior studies of dengue exposure in urban populations with similar characteristics. This prevalence was chosen to ensure adequate precision in the estimates, particularly in the context of heterogeneous transmission dynamics observed in Buenos Aires. We now added the following statement : The sample size estimation assumed an anticipated seroprevalence of 10%, based on historical data and prior studies of dengue exposure in urban populations with similar characteristics .The sample size was calculated to achieve a power of 90% with a 5% margin of error and a design effect of 1.5 to account for the complex sampling design. Based on these parameters, the required sample size was estimated at 1,487 households. This number was subsequently adjusted to 3,000 dwellings to account for potential non-response. (lines 208 to 214).

**Comment #5**: Power Calculation: The manuscript mentions a power of 90% used in the sample size calculation. However, typical sample size formulas focus on the significance level (α) rather than statistical power. Could the authors clarify how power was considered in the calculation?

**Response #5**: The sample size calculation incorporated a power of 90% (1 - β), which represents the probability of correctly rejecting the null hypothesis (i.e., detecting a true seroprevalence of dengue antibodies if it exists). While it is true that many sample size formulas emphasize the significance level (α), incorporating power is critical in ensuring sufficient sample size to achieve reliable estimates in population-based studies. In this context, power was used to minimize the risk of Type II errors, particularly given the expected variability in seroprevalence across different subpopulations in Buenos Aires. To account for the multi-stage sampling design, a design effect (DEFF) of 1.5 was applied, and the resulting sample size was adjusted to ensure sufficient power (90%) to detect seroprevalence across strata. This calculation provided an initial estimate of 1,487 households, which was increased to 3,000 dwellings to mitigate non-response.

**Comment #6**: Adjustment for Assay Sensitivity and Specificity: The dengue seroprevalence was determined using a diagnostic test with a sensitivity of 95.8% and specificity of 96.1%. Given that the specificity is less than 100%, it would be prudent to adjust the observed seroprevalence to account for assay characteristics. Based on these values, the adjusted prevalence would be approximately 5.3%. This adjustment should be incorporated into the results.

**Response #6**: The diagnostic assay used in this study has a high specificity (96.1%), minimizing the potential for false positives. Additionally, the observed seroprevalence of 8.1% is consistent with expectations for dengue exposure in Buenos Aires and is not low enough to warrant significant concern over the impact of false positives. Adjusting the prevalence in this context could result in an underestimation of true seroprevalence, particularly given the high performance of the assay. Furthermore, the rapid test results were validated through confirmatory testing using an ELFA assay, which demonstrated 100% concordance for the positive samples. This provides strong support for the validity of the unadjusted prevalence estimate.

**Comment #7**: Low Participation Rate and Generalizability: The participation rate in the study was notably low, with only 26% of the sampled population participating. This raises concerns about the generalizability of the findings. Can the authors provide any data to demonstrate that the characteristics of those who participated in the study were not significantly different from those who refused, particularly with respect to factors associated with dengue seropositivity?

**Response #7**: We acknowledge that the participation rate was lower than desired, which is a common challenge in population-based surveys, particularly in urban settings like Buenos Aires. To address this, we applied post-stratification weights through calibration by fixed margins, incorporating auxiliary data on age, sex, and geographic distribution from the Buenos Aires Directorate General of Statistics and Censuses (DGESYC) and the 2022 Annual Household Survey. This ensured that the weighted estimates reflected the demographic and geographic characteristics of the total adult population, reducing the risk of non-response bias. Unfortunately, no detailed data on the characteristics of individuals who refused to participate were collected, as is typical in survey research. However, the auxiliary data used for calibration allowed us to align the sample with the population distribution, which mitigates the impact of potential differences between participants and non-participants. Additionally, the stratified, probabilistic design ensured representation across diverse socio-economic strata, including both slum and non-slum areas. We added a paragraph under Limitations: The low participation rate (26%) introduces the possibility of non-response bias. Although calibration by fixed margins was applied to mitigate this issue, the lack of data on non-participants limits our ability to directly assess differences between participants and those who refused to participate (lines 428 to 431).

**Comment #8**: Early Termination of Participant Recruitment: The manuscript mentions that participant recruitment had to be stopped earlier than planned (Line 271). Could the authors provide additional details on why recruitment was halted prematurely?

**Response #8**: Participant recruitment was halted in December 2023 due to the onset of an unprecedented dengue outbreak in Buenos Aires. Continuing recruitment during this outbreak would have introduced a mix of pre-outbreak and intra-outbreak results, potentially confounding the study’s objectives. The primary aim of the study was to estimate dengue seroprevalence immediately prior to the outbreak, and including participants recruited during the outbreak would have undermined the conceptual clarity and temporal consistency of the findings. Despite the early termination, the study successfully captured pre-outbreak data from 804 participants, representing a substantial portion of the planned sample size. Calibration techniques were applied to ensure that the results were representative of the city’s adult population, minimizing the impact of the reduced sample size on generalizability. Please note that this is addressed in the limitations section of our manuscript (lines 420 to 424)

**Comment #9**: Inconsistency in Number of Positive Cases: On page 8, it is stated that 193,707 people tested positive for dengue (Line 173-177). However, earlier in the manuscript (Line 170), it is mentioned that only 804 interviews were conducted. This inconsistency needs clarification. Were the numbers based on extrapolation or actual testing?

**Response #9**: The number of 193,707 people testing positive for dengue antibodies refers to a weighted estimate extrapolated to the total adult population of Buenos Aires (2,386,388 individuals). This estimate was derived from the observed data collected during the survey, which included 804 participants and 71 positive cases. The weighted estimate was calculated using post-stratification weights to ensure representativeness of the entire population. This extrapolation is a standard practice in population-based probabilistic surveys. We now modified the manuscript accordingly: (please see previous response and new table 1)

**Comment #10**: Confirmatory Testing: How many of those who tested positive by the rapid test underwent confirmatory testing? This information should be included in the results section. Furthermore, the discussion mentions that only a small proportion confirmed their positive status. It would be helpful to quantify this

**Response #10**: Of the 71 participants who tested positive by the rapid test, 27 (38%) agreed to undergo confirmatory testing using the ELFA assay. Among these, all 27 results were confirmed as positive, demonstrating 100% concordance between the rapid test and the confirmatory assay. We now added in the Results section the following statement: Of the 71 participants who tested positive by the rapid test, 27 underwent confirmatory testing with the ELFA assay, all of which were confirmed as positive (100% concordance) (lines 284 to 287).

The limited proportion of participants undergoing confirmatory testing was primarily due to logistical constraints and participant non-compliance with follow-up appointments. These limitations have been acknowledged in the manuscript: The proportion of participants who underwent confirmatory testing was limited (27/71), which may affect the generalizability of the confirmatory results. However, the 100% concordance observed supports the reliability of the rapid test in this context. (lines 435 to 438).

**Comment #11**: Sensitivity and Specificity of Confirmatory Test: What were the sensitivity and specificity of the confirmatory test used in this study? Additionally, could the authors explain the rationale for conducting confirmatory testing?

**Response #11**: The confirmatory test used in this study was the VIDAS® Anti-Dengue IgG assay (bioMérieux), a qualitative automated immunoassay based on the ELFA (enzyme-linked fluorescence assay) technique. According to the manufacturer, the sensitivity of the assay is 94.3% (95% CI: 87.2–98.1%), and its specificity is 99.1% (95% CI: 97.7–99.7%). These high performance parameters make it suitable for confirmatory testing of IgG antibodies against dengue virus. he primary purpose of confirmatory testing was to validate the results of the rapid diagnostic test, which, despite its high sensitivity (95.8%) and specificity (96.1%), has inherent limitations as a point-of-care assay. The confirmatory test was selected for its superior specificity, which reduces the likelihood of false positives, and its semi-quantitative capability, which allows for a more robust validation of antibody presence. Conducting confirmatory testing provided additional confidence in the validity of the seroprevalence findings.

We added the following statement in the Method section: The VIDAS® Anti-Dengue IgG assay was selected for confirmation due to its higher specificity (99.1%) and semi-quantitative design, which complements the qualitative nature of the rapid test (lines 254 to 256).

**Comment #12**: Presentation of Factors Associated with Seropositivity: It would be beneficial to present the factors associated with dengue seropositivity in a table format for clearer interpretation. Interestingly, the confidence intervals for the odds ratios (ORs) appear to be very narrow. Did the authors compare the characteristics of those who tested positive and negative directly, or were these associations derived from the entire population based on extrapolated data?

**Response #12**: Thank you for your comment regarding the presentation of factors associated with dengue seropositivity. Below, we address your suggestions and provide clarification on the methodology used to derive the associations and the narrow confidence intervals observed.

To identify factors associated with dengue seropositivity, multivariable logistic regression models were applied, given that the outcome variable (seropositivity) is binary. Independent variables included sex, age groups, household size, living in slum areas, and a history of dengue within the household. The results are presented as odds ratios (ORs) with 95% confidence intervals (CIs) (lines 309 to 317).

The factors associated with dengue seropositivity are shown in a new table 2 (line 492). Females were more likely to test positive compared to males (OR = 1.442, 95% CI: 1.429–1.456). Living in a slum area was associated with higher seropositivity (OR = 1.151, 95% CI: 1.133–1.169), as was an increasing number of household members (OR = 1.039, 95% CI: 1.036–1.043). Age was inversely associated with seropositivity, with individuals aged 18–35 years being significantly more likely to test positive (OR = 2.604, 95% CI: 2.567–2.641) compared to those aged 65 years and older (reference group). Finally, having a household history of dengue was strongly associated with seropositivity (OR = 5.762, 95% CI: 5.709–5.817). See new table 2 and (lines 309 to 317).

|  | OR | Lower 95% CI | Upper 95% CI |
| --- | --- | --- | --- |
| Female sex | 1,442 | 1,429 | 1,456 |
| Living in a slum area | 1,151 | 1,133 | 1,169 |
| Number of household residents | 1,039 | 1,036 | 1,043 |
| Age ≥65 years | 1 |  |  |
| Age ≥50 y <65 years | 0,91 | 0,894 | 0,926 |
| Age ≥35 y <50 years | 0,875 | 0,861 | 0,889 |
| Age ≥18 y <35 years | 2,604 | 2,567 | 2,641 |
| History of dengue in the household | 5,762 | 5,709 | 5,817 |

We also added a brief comment under into the Discussion section: These results highlight important associations with dengue seropositivity, such as higher rates among younger adults, females, individuals living in slum areas, and those with household members previously infected by dengue. However, it is important to note that this study was designed primarily to describe seroprevalence, and these associations should be interpreted cautiously in the context of this descriptive study (lines 372 to 376).

We sincerely thank the reviewers for their thorough and insightful comments on our manuscript. We have carefully reviewed and addressed each of the points raised, ensuring that every suggestion was considered with the utmost attention. We firmly believe that the manuscript has been substantially improved thanks to the constructive feedback provided. Your comments have helped us clarify key aspects of the study, refine our analyses, and strengthen the overall presentation of our findings. We deeply appreciate the time and effort you have devoted to this review process
