# Supplementary material for: Prevalence of antibodies against dengue virus in the city of Buenos Aires: Results of a probabilistic population survey
Source: PLoS Negl Trop Dis. 2025 Feb 18;19(2):e0012886. doi: 10.1371/journal.pntd.0012886 (PMC11856294; doi:10.1371/journal.pntd.0012886)
Supplement: Attachment — Submitted filename: Response to reviewer.docx [file pntd.0012886.s003.docx]

**Responses to reviewer**

**Comment #1:**

“While the rationale for this study is justified by efforts to better understand the epidemiology of dengue in Argentina, the reviewer’s comment to the authors to provide a brief description of the burden of this disease is not addressed in the Introduction section, and therefore, the author’s implied statement (line 24) that dengue is a rapidly growing health issue in Argentina based only on outbreaks does not support dengue as being a health issue. Why estimates of the disease burden is not presented is not understood, but would help to justify the rationale for conducting the study. That estimates were available is supported by the information presented in the Discussion section, lines 343 -345 that data from the latest epidemiological bulletin indicated that 68% of cases were from DEN-2, while 32% were caused by DEN-1. Also according to lines 293-297, “In Buenos Aires, using reported case data for those aged ≥18 years, the number of affected individuals was 16,138,294 (91% serologically confirmed) as of November 2023, generating an estimated prevalence of approximately 682 cases per 100,000 inhabitants. As stated, inclusion of this information in the Introduction would help to justify the rationale for conducting this study and for supporting the statement that dengue is a rapidly growing health issue in Argentina. Also, of relevance to including information of the disease burden, in lines 103-105, it is stated that “However, recommendations for implementing a vaccination program must consider many factors, among which the burden of infection is one of the most important. Since the majority of dengue infection are asymptomatic or mild, again, this should read the burden of disease and not the burden of infection”.

**Response #1:**

We have revised the Introduction to include a summary of the burden of disease in Argentina, based on data from the National Epidemiological Bulletin and those previously presented in the Discussion, as well as data from the Pan American Health Organization (**lines 79 to 90**). Additionally, we include in this response a figure (only for the reviewer) based on the cases reported by PAHO and the epidemiological bulletin to illustrate the case dynamics. Please note that the 2023 outbreak began gradually in the last epidemiological week of 2022 and extended until epidemiological week 24 of 2023. After that, no further cases were reported until approximately week 48 of 2023 **(mentioned in new lines 79-90)**. We believe this is important to show the reviewer (and have added it to the Introduction as requested), both to demonstrate the dynamics of infections and to explain and contextualize the end of recruitment on November 30, 2023, which coincided with epidemiological week 48. Please note that the study presented in this manuscript was conducted during epidemiological weeks 34 to 48 of 2023, corresponding to the period from August 19, 2023, to November 30, 2023). Please note that our study was conducted during the period marked between the dashed lines in the year 2023.

Additionally, we have corrected the wording in lines 103-105, replacing 'burden of infection' with 'burden of disease,' in line with the reviewer’s observation (**lines 125**).

**Comment #2:**

“While the implied purpose of serosurvey in this study was to determine if a dengue vaccination campaign was needed, the specific purpose of the study is vague, for example, it is stated in lines 112-116 that “Considering the heterogeneity in the distribution of dengue disease both between and within countries, the WHO Strategic Advisory Group of Experts (SAGE) recommends the implementation of local epidemiology plans (5). In this manuscript, we report the first determination of dengue seroprevalence in Argentina, particularly in the City of Buenos Aires, using a probabilistic approach”. Does this WHO plan recommend this seroprevalence study, and if so, it is not clear how this study will contribute to the understanding of dengue disease.
The study site could be improved by more information on the targeted population, for example, a breakdown of the total population of the slum section versus other socially and demographic sectors of the city, such as occupations, water use practices, sanitation,, health care, and mosquito control services. As described, the information provided is that general private households’ frame, comprises approximately 93% of the dwellings in Buenos Aires, while the second frame, called the “Informal Popular Neighborhoods” frame, that included the dwellings in the city’s slums”

**Response#2**

The study’s objective was defined before its initiation (the study number and objectives can be verified on the ClinicalTrials.gov website) and is stated in the first sentence of the abstract. Our study aimed to measure the seroprevalence of dengue antibodies in adult residents of Buenos Aires. Assessing seroprevalence requires a comprehensive study that goes beyond case reporting.

The burden of dengue disease encompasses more than the number of symptomatic cases, as it also includes the potential reservoir of infected individuals, regardless of symptomatology or official reporting. It is important to recognize that this burden extends beyond direct costs such as hospitalizations, treatments, and lost workdays, and includes broader public health implications that require a comprehensive understanding of the population's seroprevalence

Rather than estimating how many individuals had symptoms, our study aimed to determine how many people in Buenos Aires have dengue antibodies. This aligns with WHO recommendations, which encourage each country and city to establish this metric as part of their epidemiological surveillance efforts. Although this study was not designed under a specific WHO mandate, our goal was to contribute to the understanding of dengue epidemiology in a complex urban setting such as Buenos Aires. This approach allows for the exploration of inequalities in infection risk across different socioeconomic sectors and environmental contexts, which is essential for implementing targeted interventions.

Regarding the information on the target population, we have expanded the description of the sampling frameworks used (**lines 101 to 111**). These include the general private housing framework, which covers approximately 93% of households in Buenos Aires, and the Informal Popular Neighborhoods framework, which includes households in the city’s slums.

**Comment #3**
“The comment by the reviewer as to why only adults 18 years of age and older were enrolled in this study was addressed by the authors (lines 391-401), but the reason should be discussed regarding the potential impact of the exclusion of the younger ages on the validity of the results as being representative of the entire population.as stated by the authors that the results may have limited generalizability to younger age groups, and therefore interpreted by the reviewer as posing the critical question regarding the repeated claim that the finding were representative of the entire population. According to the WHO’s policies, estimates of infection/disease burden for making a decision on vaccination campaigns should be based on the results of studies involving school-aged children, 5-18 years of age.”

**Response #3**

We have further emphasized the limitation pointed out by the reviewer. Although this was already stated, we have added an explicit statement: “Moreover, the results of this study cannot be automatically extended to the entire population of Buenos Aires but only to individuals aged 18 years and older. This also poses the challenge that vaccination recommendations are typically based on seroprevalence levels among those aged 5 to 18 years. Although seroprevalence in children tends to be higher than in adults, the likelihood that seroprevalence in children from Buenos Aires would fall within recommendation thresholds is low” (lines 455 to 461).

**Comment #4**

“Line 412 The design of the sampling method did not appear consider the well documented observation that dengue virus transmission is known to occur in spatial clusters. On the basis of this observation and the very low number of samples and especially the low seroprevalence, especially by the limited number per age groups need to be addressed regarding the validity of the representation of the total population and addressed as a possible limitation of the outcome of the study”.

**Response #4**

This comment has already been addressed, and the text highlighted by the reviewer was included following a previous review. As mentioned in the manuscript, we acknowledge that dengue transmission occurs in spatial clusters, and this phenomenon could have influenced the results of our study. To address this challenge, we employed a stratified sampling design and weighted the data to reflect the population distribution. However, we are aware that the limited sample sizes per age group and the low seroprevalence values might have affected the representativeness of our findings.

We have now placed additional emphasis on this limitation to make it explicit, recognizing that future research could benefit from integrating geospatial models to better assess the spatial heterogeneity in dengue transmission.

We added the following: 'It is important to recognize that dengue transmission typically occurs in spatial clusters, a phenomenon that was not fully addressed in this study. While we used a stratified sampling design and applied weights to reflect the population distribution, these strategies do not fully capture the spatial dynamics of transmission. Additionally, the limited number of samples per age group and the low seroprevalence values might have reduced the representativeness of the results. Future studies should consider the use of geospatial approaches to better model these dynamics and ensure a more accurate representation of dengue transmission (lines 479-487).

**Comment #5**

“The reviewer suggest that in addition to the city and country name of the assay manufacturers, and if available, publication(s) in support of the sensitivity and specificity of the assay data be added to the manuscript. Also, include the dilution made automatically and the reason for collecting blood samples a second time. Was there agreement of the results of all samples between the 2 assays. Were the manufacturer’s data on sensitivity and specificity validated and if so, describe.”

**Response #5**

We have added the country of origin of the assay manufacturers used: the rapid test was produced by Laboratorios JAYOR SRL (Argentina), and the confirmatory VIDAS Anti-Dengue IgG assay by bioMérieux (France) (lines 226 and 236). The sensitivity and specificity data reported in the manuscript correspond to the values provided by the manufacturers and compared against ELISA in both cases. We did not perform an independent validation of these values.

The VIDAS Anti-Dengue IgG assay includes an automatic dilution step performed by the VIDAS system, as mentioned in the manuscript (line 238). This means that the VIDAS assay automatically dilutes the serum sample as part of its process, without requiring manual handling. According to the manufacturer, this dilution is 1:400 and is performed internally by the VIDAS system, ensuring the accuracy and standardization of the procedure without manual intervention. We added this information (lines 242-244)

The second blood sample collection was performed only on individuals with positive IgG results in the rapid test (already described in lines 233-234). This allowed us to confirm the results using a method with higher specificity and provide cross-validation between the two assays.

Regarding the comment on result consistency, please note that this information is already present in the revised manuscript (lines 273 to 276), where it states: “Of the 71 participants who tested positive by the rapid test, 27 underwent confirmatory testing with the ELFA assay, all of which were confirmed as positive (100% concordance)”.

**Comment #6:**

Since the outcome of DENV infection cannot be determined based seroprevalence estimates, the reviewer suggest that an explanation be provided regarding the statement that serial population based seroprevalence studies can serve as a robust system for assessing disease burden.

**Response #7:**

It is important to clarify that the objective of this study was to determine the seroprevalence of dengue virus antibodies among adults residing in the city of Buenos Aires, rather than estimating the number of symptomatic dengue cases. Seroprevalence studies serve as a complementary tool to epidemiological surveillance based on the notification of symptomatic cases and the use of mathematical models, providing a more comprehensive view of virus circulation in the population. We acknowledge that no single method is sufficient to precisely determine disease burden or guide health policy decisions. While seroprevalence studies are necessary, they must be interpreted alongside other epidemiological and modelling data to inform public health decisions effectively. To clarify this point, we have included at the very beginning of the discussion section (lines 314 to 321)

**Comment #8:**

According to the authors, the disparity between the reported symptomatic cases of 682 per 100,000 reported based on assumed passive surveillance as of November 2023 in Buenos Aires and the estimate 9,050 cases per 100,000 observed in this study was because many of the dengue virus infections detected in the seroprevalence study were asymptomatic. While many dengue virus infections are asymptomatic, how was estimate of symptomatic cases based on seroprevalence data determined?

**Response #8:**

This study did not directly estimate the number of symptomatic cases based on seroprevalence data. The comparison between the reported case incidence and the estimated seroprevalence was used solely to illustrate the difference between infections recorded through passive surveillance and the total number of infections, including undiagnosed or asymptomatic cases. While the literature suggests that a significant proportion of dengue infections are asymptomatic, accurately estimating the symptomatic disease burden requires additional studies that combine seroprevalence data with longitudinal information on the proportion of symptomatic infections in the population (please see previous response #7 and changes made on manuscript accordingly).

**Comment #9:**

Lines 319-325 According to the results, “The age distribution of DENV antibody-positive participants showed a higher prevalence in younger adults as compared to the older age groups”. As implied by the authors, this observation is not consistent with the existing understanding of the epidemiology of dengue even in non-hyper-endemic areas, and it is not understood how according to the authors, the concentration of more recent outbreaks could occur among the younger, more mobile populations. Furthermore, it is not understood how the observation “underscores the need for targeted prevention strategies and ongoing surveillance to monitor shifting transmission dynamics”.) Lines 26-28

**Response #9:**

While dengue epidemiology is often associated with a higher accumulation of infections among older adults in low-SDI regions, several studies have indicated that in urban areas with recent outbreaks, incidence may be higher among young adults. According to the Global Burden of Disease Study, the global incidence of dengue is highest among adolescents and young adults (10–25 years), followed by young children (5–10 years) and adults aged 25–49 years, with an increase in older adults only in low and low-middle SDI regions (GBD, 2019). (New reference #13 -line 557-559: Du M, Jing W, Liu M, et al. The Global Trends and Regional Differences in Incidence of Dengue Infection from 1990 to 2019: An Analysis from the Global Burden of Disease Study 2019. Infect Dis Ther. 2021;10:1625-1643.).

In this context, we consider that the concentration of recent outbreaks in younger, more mobile populations may be related to differential exposure factors in urban areas, such as mobility, occupational activities, and increased contact in transmission-prone settings. As mentioned in the manuscript, this observation highlights the need for targeted prevention strategies and continuous surveillance to monitor changes in dengue transmission, in line with previous studies on dengue epidemiology in different regions.

"We have added a further explanation in the manuscript (lines 357 to 363)."

**Comment #10**

As a major weakness of this study that is not mentioned in the results section, but is presented in the discussion section that participant recruitment had to be stopped earlier than planned. According to the authors, recruitment did not begin until September, 2023 and ended immediately before the subsequent dengue epidemic in early 2024, the specific date of the survey being from September 21 until December 1, 2023.The reason according to the authors was because of the unusually aggressive dengue outbreak recorded across Argentina since December 2023, which is not understood, is this year correct? Considering that recruitment began in September 2023, the collected data reflected the prevalence status before or during the beginning of the 2024 new epidemic. According to the authors, “Under these conditions, it was decided to stop recruitment and take an estimate of dengue seroprevalence before the epidemic in early 2024 in Argentina”. The reviewer’s is led to believe that the enrollment of only 808 of the planned number of 2,998 participants was because of the very low enrollment rate, but then according to the authors, the reason was because of the study was aborted because of the dengue epidemic. Also, with the largest ongoing outbreak ever recorded in Argentina, the authors need to provide an explanation for the very low dengue IgG and IgM antibody prevalent rate, especially only a 1.5% IgM antibody rate that would not appear to adequately reflect the recent infection or during the past 2 or more months the estimated duration of IgM antibody.

**Response #10**

Please see also response #1.

As indicated in the text, participant recruitment was conducted between September and December 2023, corresponding to epidemiological weeks 34 to 48 of that year.

Please note that the planned sample size (declared since the first submission of the article and currently present in lines 207 to 210 of the tracked-changes manuscript) is not 2,998, as mentioned by the reviewer, but 1,487. The number cited by the reviewer refers to the total number of households approached, which was based on an estimated non-response rate of approximately 50%.

We believe that the figure we provided (response #1) (which is based on publicly available data from the PAHO website) and the explanations given in the manuscript should provide sufficient context for understanding the rationale behind the study's early termination.

In the figure to the referee based on the data reported by PAHO and the National Epidemiological Bulletin is clear that no active dengue transmission occurred in Buenos Aires between epidemiological week 24 and week 48 of 2023, the period in which the study was conducted. Regarding the IgM prevalence (1.5%), this result is consistent with the low viral circulation period during which the study was conducted.

For additional clarity, we have reinforced these explanations in the manuscript (lines 258 to 260 and 426 to 432).

**Comment #11**

In the discussion section it is stated that “Although dengue is a notifiable disease, it is likely that the number of reported cases underestimates the disease burden due to the presence of asymptomatic or mildly symptomatic cases, as well as a tendency for underreporting communicable diseases”. It is not clear as to the meaning of the latter sentence, as asymptomatic cases would not contribute to the burden of disease, under-reporting would lessen the estimated burden of disease, but is there evidence for under-reporting of cases.

**Response #11**

See also response “2.

**Comment #12**

Firstly, participant recruitment had to be stopped earlier than planned. The main reason is not clear, but understood to be because of the unusually aggressive dengue outbreak recorded across Argentina since December 2023. Considering that recruitment began in September 2023, the collected data reflected the prevalence status before the new epidemic. Under these conditions, it was decided to stop recruitment and take an estimate of dengue seroprevalence before the most recent epidemic in early 2024 in Argentina. Ongoing surveys will provide information on how this situation may have changed. As understood, surveillance was terminated December 1, 2023, what ongoing surveillance is being referred too?

**Response #12**

Please see response # 1 and #10.

When we refer to "ongoing surveys," we mean ongoing epidemiological surveillance carried out by health authorities and other population-based studies that continue to monitor the evolution of the 2024 epidemic. We are not referring to a continuation of our study but rather to additional efforts to assess how the situation has changed following the new outbreak.

For clarity, we have revised this section of the manuscript (lines 433-438)

**Comment #13**

Additionally, the non-response rate was higher than expected. As stated in lines 233- 234 and lines 246- 248, “Out of the 2,998 selected households, 804 individuals participated in the study, resulting in a response rate of only 26.8%”. The low participation rate introduces the possibility of non-response bias. Although calibration by fixed margins was applied to mitigate this issue, the lack of data on non-participants limits our ability to directly assess differences between participants and those who refused to participate. The very low enrollment of 804 participants resulted in only about 200 participants per age group with no assurance that these participants were representative of the entire population of 2.38 million people.

**Response #13**

Please see response #10.

The possibility of non-response bias has already been acknowledged and discussed in the manuscript (lines 440-447) As stated, the participation rate was lower than expected, which could introduce selection bias. To mitigate this issue, we applied calibration by fixed margins, a widely used method in population-based surveys to improve the representativeness of the sample in relation to the target population.

While response rates in population-based studies involving biological sample collection can vary, the methodology used in this study follows rigorous epidemiological standards. This study does not aim to replace epidemiological surveillance or make absolute inferences about the entire population of Buenos Aires but rather to provide an estimate based on a representative sample within its methodological constraints, which have been clearly outlined and discussed in the manuscript.

**Comment #14**

The proportion of participants who underwent confirmatory testing was very limited (27/71), which may affect the generalizability of the confirmatory results. However, the 100% concordance observed supports the reliability of the rapid test in this context. With the interruption of the study plans, especially the substantial reduction in the number of enrolled participants, it is not clear as to how reliable the estimated prevalence rate was for the entire city and the observations to confirm or refute the validity of the extent of being representative are not presented in this study .

**Response #14**

We would like to reiterate that the 100% concordance observed between the rapid test and the confirmatory assay supports the reliability of the test used in this study. While the proportion of participants who underwent confirmatory testing was limited (27/71), this analysis was not intended to validate the rapid test, but rather to provide an additional level of certainty in the interpretation of the results.

Regarding the representativeness of the estimated seroprevalence, we emphasize that standard epidemiological approaches were used, including calibration by fixed margins, to improve the correspondence between the sample and the target population. As in any population-based study, there is no absolute metric for representativeness, but the methodology has been applied transparently, and its limitations have been thoroughly discussed in the manuscript.

The objective of this study was to generate an estimate of dengue seroprevalence in Buenos Aires within the framework of the methodological limitations previously outlined. This estimate is not intended to replace other sources of epidemiological surveillance, but rather to provide complementary information based on a population-based approach.

**Comment #15**

The exclusion of the under 18 years of age in the study is likely to have resulted in a bias that is not accounted for regarding the overall prevalence of dengue virus antibody in the entire population of the city. An explanation is provided for the exclusion of under the age of 18 year, and while stating that the results may have limited generalizability to younger age groups, and therefore raises critical question regarding the repeated claim that the finding were representative of the entire population.

**Response #15**

This has already been addressed in comment and response #3. Please refer to that response as well as lines 455 to 461 of the revised manuscript.

**Comment #16**

The validity of using an approach based on weighted estimates for dengue virus seroprevalence could be biased because this approach would assumed that the risk of dengue infection is homogeneous, an assumption that is not clearly addressed in this study, for example variation in the distribution of Aedes aegypti, that is a major determinant of temporal and spatial distribution of dengue virus infection, and that the slum population differed from the other 93% of the population is not supported by any relevant information in regards to the potential

risk of dengue virus infection.

**Response #16**

We acknowledge that the distribution of dengue infection risk is not homogeneous within a city. However, the use of weighted estimates does not assume homogeneity in infection risk but rather allows the sample to be adjusted to better reflect the known population distribution. Calibration by fixed margins is a standard epidemiological technique used to improve representativeness in population-based studies, although it does not completely eliminate the possibility of spatial biases.

We understand that the distribution of *Aedes aegypti* is a key determinant of dengue transmission. In this regard, we have incorporated information on the sociodemographic differences between the slum population and the rest of the city, as factors such as population density, access to basic services, and housing conditions may influence infection risk. This information can be found in lines 101-111 of the manuscript as you requested.

While the primary objective of this study was not to model the spatial distribution of risk, we consider our findings a valuable starting point for future research incorporating geospatial models and more detailed analyses of environmental and entomological factors

**Comment #17**

With the largest ongoing outbreak ever recorded in Argentina during 2023, what is the explanation for the very low dengue IgG antibody (8%) and IgM antibody prevalent rate, especially the 1.5% IgM rate disease, a rate that indicate a low risk for DENV infection. Also, the conclusions that the results of this study showed a modest burden of dengue in the adult population of Buenos Aires immediately before the 2024 outbreak is not supported as seroprevalence cannot be used to estimate the burden of dengue disease.

**Response #17**

Please see also previous responses. The results are consistent with the period in which the survey was conducted and the epidemiological dynamics of dengue in Buenos Aires. The presence of IgM reflects recent infections, and its positivity lasts for a limited period, typically between 2 and 3 months. Since the study was conducted during a period of no active transmission (epidemiological weeks 34 to 48 of 2023), a low proportion of IgM-positive individuals was expected, as the 2023 epidemic had ended months earlier and the new epidemic had not yet begun. On the other hand, the observed IgG seroprevalence is not inconsistent with a previous epidemic outbreak.

Regarding the disease burden, seroprevalence does not aim to measure the clinical burden of dengue but rather provides an estimate of the number of individuals who have developed antibodies, including asymptomatic or unreported infections As we previously mentioned (lines 314-321) this information is complementary to epidemiological surveillance and other public health metrics used to assess the impact of dengue.

**Specific comments**

**Comment #18 to 21**

C18: Aedes aegypti and Aedes albopictus need to be Italicized throughout the manuscript.

R18: The have been italicized throughout the manuscript.

C19: In lines 21 -23 it is stated that, “The VIDAS Anti-Dengue IgG assay was selected for confirmation due to its higher specificity (99.1%) and semi-quantitative design, which complements the qualitative nature of the rapid test”. As such, Anti-Dengue IgG should read Dengue IgG antibody as Anti is redundant and should include antibody to be more specific to replace Anti

R19: Anti-Dengue IgG' has been replaced with 'Dengue IgG antibody' to improve clarity and consistency in terminology (**line 145, 224-225, 236, 246)**.

C20: In lines 126-132, as examples of the misuse of grammar as well as elsewhere in the manuscript, the past tense should be used as follows: “Consequently, the sample results (add were and delete are) valid representations of the entire city. This approach (add allowed and delete allows) for the inclusion of diverse subpopulations by dividing the sampling frame into strata based on relevant criteria, such as income levels and geographic areas. By employing multi-stage sampling, we (add optimized and delete optimize) resource allocation while maintaining statistical reliability, as each stage progressively (add narrowed and delete narrows) the selection from broader units (e.g., census tracts) to individual households and participants. This methodology (add reduced and delete reduce) sampling bias and (add enhanced and delete enhances) enhances the validity of results for an urban area as complex and diverse as Buenos Aires.

R20: We have revised the manuscript to ensure proper use of past tense

C21: In lines 403-405 it is stated that “Most DENV infections are asymptomatic, and antibody detection reflects prior exposure rather than current infection rates”. Suggest this sentence be revised to read, “Most DENV infections are asymptomatic, and antibody detection reflects prior infections rather than current infection rates”.

R21: We have modified the manuscript accordingly.
